# Microbial Communities across Global Marine Basins Show Important Compositional Similarities by Depth

**DOI:** 10.1128/mBio.01448-20

**Published:** 2020-08-18

**Authors:** John I. Miller, Stephen Techtmann, Dominique Joyner, Nagissa Mahmoudi, Julian Fortney, James A. Fordyce, Nargiz GaraJayeva, Faig S. Askerov, Claudio Cravid, Maarten Kuijper, Oliver Pelz, Terry C. Hazen

**Affiliations:** a Bredesen Center, University of Tennessee, Knoxville, Tennessee, USA; b Department of Civil & Environmental Engineering, University of Tennessee, Knoxville, Tennessee, USA; c Department of Ecology & Evolutionary Biology, University of Tennessee, Knoxville, Tennessee, USA; d BP International, Sunbury on Thames, United Kingdom; CEH-Oxford

**Keywords:** genomics, marine microbiology, microbial communities, microbial ecology, oil biodegradation

## Abstract

The environmental surveys following the 2010 Deepwater Horizon (DWH) spill identified a variety of hydrocarbon-degrading microorganisms, and laboratory studies with field-collected water samples then demonstrated faster-than-expected hydrocarbon biodegradation rates at 5°C. Knowledge about microbial community composition, diversity, and functional metabolic capabilities aids in understanding and predicting petroleum biodegradation by microbial communities *in situ* and is therefore an important component of the petroleum spill response decision-making process. This study investigates the taxonomic composition of microbial communities in six different global basins where petroleum and gas activities occur. Shallow-water communities were strikingly similar across basins, while deep-water communities tended to show subclusters by basin, with communities from the epipelagic, mesopelagic, and bathypelagic zones sometimes appearing within the same cluster. Microbial taxa that were enriched in the water column in the Gulf of Mexico following the DWH spill were found across marine basins. Several hydrocarbon-degrading genera (e.g., *Actinobacteria, Pseudomonas*, and *Rhodobacteriacea*) were common across all basins. Other genera such as *Pseudoalteromonas* and *Oleibacter* were highly enriched in specific basins.

## Abstract (precis)

Marine microbial communities are a vital component of global carbon cycling, and numerous studies have shown that populations of petroleum-degrading bacteria are ubiquitous in the oceans. Few studies have attempted to distinguish all of the taxa that might contribute to petroleum biodegradation (including, e.g., heterotrophic and nondesignated microbes that respond positively to petroleum and microbes that grow on petroleum as the sole carbon source). This study quantifies the subpopulations of microorganisms that are expected to be involved in petroleum hydrocarbon biodegradation, which is important information during the decision-making process in the event of a petroleum spill accident.

## INTRODUCTION

The environmental sampling and studies following the 2010 Deepwater Horizon (DWH) accident in the Gulf of Mexico demonstrated faster-than-expected hydrocarbon biodegradation rates in deep water (at 5°C) (1). This result, which was based on field and lab studies with field-collected water samples, showed hydrocarbon composition changes with distance from the DWH well blow out, and revealed a variety of hydrocarbon-degrading microorganisms (1). The indigenous microbial community contained oil-degrading microorganisms adapted to natural seeps of crude oil from reservoirs (2). Rapid oil biodegradation by these indigenous oil degraders was facilitated by a high prevalence of water-soluble constituents in the spilled crude oil (3) and by injection of subsea dispersant into the erupting oil flow (4). Furthermore, the microbial community composition and dominant taxa changed rapidly with the changing petroleum composition during the crude oil degradation and weathering processes (5).

Petroleum spills and subsequent environmental exposure can have severe, detrimental effects in the immediate release site as well as nearby ecosystems exposed to toxic levels of oil hydrocarbons, depending on the amount of petroleum released (6, 7). These incidents may occur during oil and gas exploration or production-related activities, including accidents during storage or transport (7). Traditional response methods to combat oil spills include skimming (to recover petroleum from the sea surface), shoreline cleanup, aerial and subsea application of chemical dispersants (with airplanes or vessels), *in situ* burning of floating petroleum, and biodegradation. Of these response methods, petroleum bioremediation (i.e., biodegradation by microorganisms) of petroleum can be effective while having minimal additional adverse effects on the environment (7). However, environmental conditions such as temperature, oxygen concentration, and available nutrients influence the rate and extent of petroleum bioremediation (6, 8, 9). The selection of the most appropriate response option(s) following an oil spill typically involves the consideration of many factors and trade-offs, which can seem overwhelming. A structured spill impact mitigation assessment (SIMA) process has been developed to facilitate selection of response option(s) and to support strategy development (10). The Braer accident at the Shetland Islands (1993) and the Sea Empress accident in Wales (1996) have demonstrated how different oil behaviors can be in a spill (11, 12).

Marine microbial communities are a vital component of global carbon cycling, and numerous studies have shown that populations of known oil-degrading bacteria are ubiquitous in oceanic environments (6, 13–16). Biodegradation of petroleum, which is a highly complex mixture of hydrocarbons, requires a complex community of microorganisms (1, 17–19). Knowledge about microbial community composition and diversity aids in understanding and prediction of petroleum biodegradation by microbial communities *in situ* and is therefore an important component of the SIMA oil spill response decision-making process (e.g., dispersant application to enhance oil biodegradation) (7). Availability of nutrients (e.g., nitrogen, phosphorus, potassium, and iron) and electron acceptors (e.g., available dissolved oxygen) may limit microbial growth, preventing biodegradation of petroleum (20). Characterization of the microbial community in the Gulf of Mexico following the DWH spill provided insight about succession of microbial taxa that are involved in petroleum biodegradation *in situ* (1, 5). Similar transitions in community composition have been observed in microcosm experiments following amendment of seawater samples with petroleum hydrocarbons (21, 22). These analyses support, in general, a paradigm of successive blooms of taxonomically distinct indigenous microbial populations as the oil weathers and labile components are sequentially degraded, leaving less-readily degraded components to feed subsequent blooms (2, 17, 23–27).

This study investigates the taxonomic composition of microbial communities in six different global basins where oil and gas activities occur. The following basins were investigated: the Sargasso Sea, the Angola basin, the Caspian Sea (Azerbaijan), the Great Australian Bight, and central and eastern Mediterranean Sea (Libya and Egypt, respectively). The Atlantic Ocean is divided by the midocean ridge system, which reduces current flow between the west and east basins (7, 28). Members of the *Oceanospirillaceae*, which were enriched during the Deepwater Horizon spill in the Gulf of Mexico, have been reported in the East (Angola) basin and may be important members of the community in the event of an oil spill (1, 29). In the Mediterranean Sea, North Atlantic Ocean water flows in via surface currents through the Strait of Gibraltar, while high-salinity water flows out via deep currents, beneath the inflowing North Atlantic water, making this basin an inverse estuary (7). The Caspian Sea is the largest landlocked body of water in the world. Its waters are brackish (salinity ∼1/3 of ocean seawater) and dissolved oxygen (DO) concentration decreases with depth due to infrequent deep-water renewal (30). The Great Australian Bight (GAB) lies off Australia’s southern coast and receives input from the Indian and Southern Oceans. These waters are well ventilated, and DO concentrations are well above hypoxic levels (7). The environments in this study are locations of petroleum exploration, production, and transport; therefore, the study of microbes and processes involved in biodegradation is essential both for oil and gas industries operating in these basins and for the governmental and regulatory bodies responsible for environmental stewardship. Both dispersal (across geographic locations and depth) and selection (due to environmental factors) are important ecological phenomena that influence microbial community composition (13, 16, 17). The primary goal of this study was to determine the relative importance of geographic location, depth, and environmental factors in shaping the taxonomic composition of these microbial communities. Special attention was given to reported petroleum-degrading genera across basins, which is important SIMA information for assessing potential fate and impacts of an oil spill event in each basin.

## RESULTS

**FIG 1:**
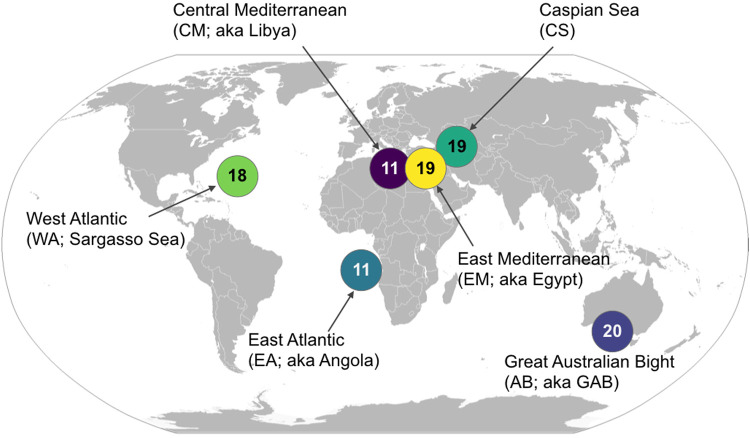
BP’s 2013–2014 environmental survey sampled along the entire water column at geographically distinct marine basins. In total, 98 seawater samples were collected from basins as indicated. At each sample site, two to four seawater samples were collected at discrete intervals evenly distributed across the entire water column for characterization of the microbial community. Environmental data were collected by connectivity, temperature, and depth (CTD) continuously to the seafloor.

**FIG 2:**
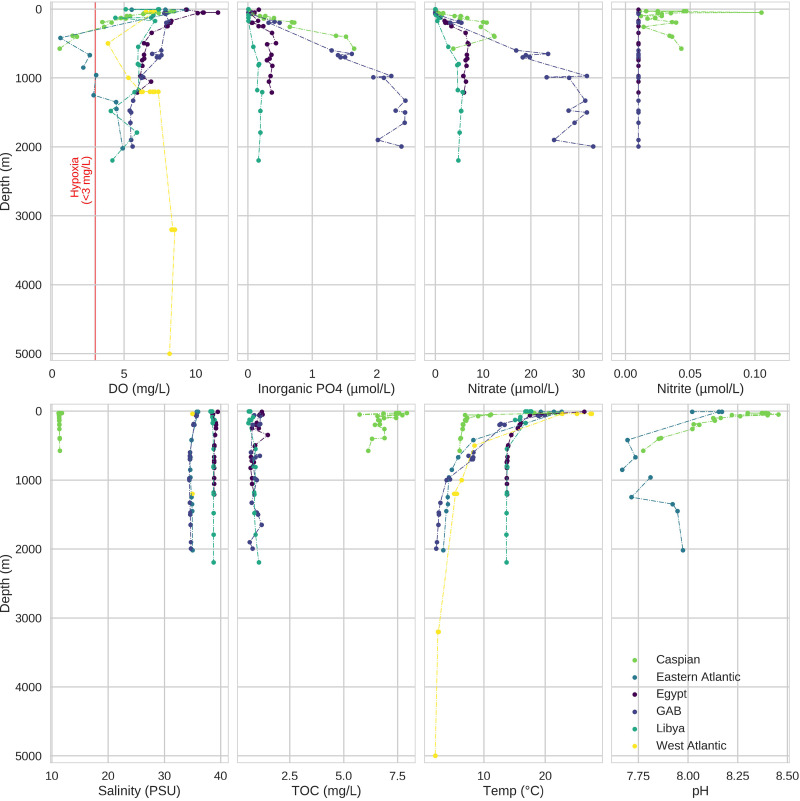
Depth profiles for environmental factors across basins. Some environmental factors fluctuated between sample sites within a single basin, while others were more consistent. For example, inorganic phosphate and nitrate in the GAB are highly variable. Within each basin, three to eight sites were sampled. Environmental data were collected at each sample site by CTD continuously to the sea floor, while seawater samples for microbial community characterization were collected at discrete intervals. Markers indicate the value of the environmental factor at the depth of the seawater sample. Data on some environmental factors were not available for all sample locations. PSU, practical salinity units: TOC, total organic carbon.

Ninety-eight samples were obtained from six marine basins: the Sargasso Sea, the Angola basin, central and east Mediterranean Sea (Libya, Egypt), the Great Australian Bight (GAB) (Australia), and the Caspian Sea (Azerbaijan; Fig. 1). Ambient environmental conditions were measured at each of the six basins (Fig. 2; see also Fig. S1 in the supplemental material). A thermocline was present at ∼50-m depth in all basins; however, temperatures in the GAB fluctuated more than other basins until nearly 1,000 m. The Sargasso Sea was the deepest basin (5,000 m) and was the only basin in this study that included samples from the abyssopelagic zone. On the other hand, the Caspian Sea was the shallowest basin (590 m), and samples were no deeper than the mesopelagic zone. All other basins in this study included samples as deep as the bathypelagic zone. The Mediterranean Sea (Libya, Egypt) was the warmest, with minimum temperature ∼15°C, while all other basins reached 8°C by 600 m. The waters of the abyssopelagic zone in the Sargasso Sea were the coldest (2.5°C).

10.1128/mBio.01448-20.1 FIG S1 Supplemental depth profiles for environmental factors across basins. Data on some environmental factors were not available for all basins; no environmental data were available for the Sargasso Sea. Download FIG S1, DOCX file, 0.4 MB. Copyright © 2020 Miller et al. 2020 Miller et al. This content is distributed under the terms of the Creative Commons Attribution 4.0 International license.

**FIG 3:**
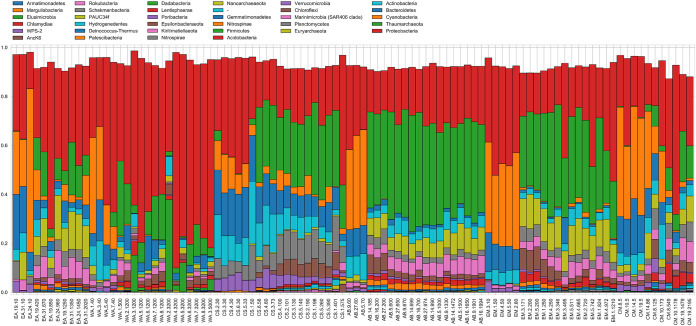
Relative abundance of phyla across basins. Cyanobacteria are enriched in shallow-water communities, while *Thaumarchaeota* and *Proteobacteria* are enriched in deep-water communities. The distribution of *Actinobacteria* and *Bacteroidetes* is more even across depths. Only OTUs that were present at >0.1% relative abundance were included in the plot.

**FIG 4:**
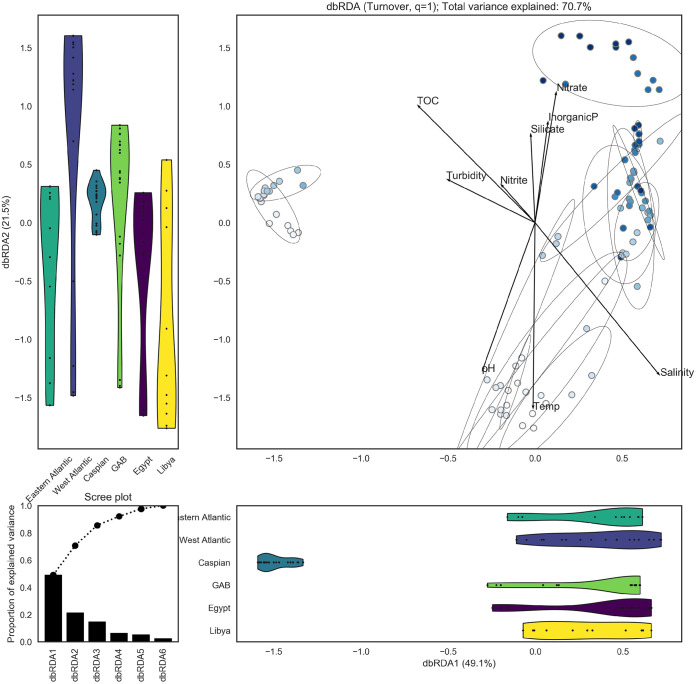
dbRDA plot illustrates dissimilarities between microbial communities. The Caspian Sea (ordination, left) is home to unique microbial communities. Overall, microbial communities are distributed along a depth gradient (ordination, darker blue markers represent deeper samples). Markers represent microbial communities, and darker blue markers indicate communities from deeper waters. Ellipses indicate 95% confidence intervals for groups of communities from the same basin and pelagic zone. Vectors indicate environmental features that were correlated with the microbial communities in the ordination space. Violin plots illustrate the distribution of microbial communities from each basin along the first (lower) and second (left) ordination components. The scree plot (bottom left) illustrates the proportion of variance explained by each of the constrained components in the ordination.

Microbial communities show important differences by depth (Fig. 3). Richness and alpha-diversity were calculated using rarefied read counts as Hill numbers *D* (effective number of operational taxonomic units [OTUs]) (Fig. S2). Hill numbers were calculated with the parameter *q* from 0 to 2; as *q* increases, rare species are given less weight and therefore contribute less toward “effective number of OTUs” (31). Significant correlations were not detected between depth and alpha-diversity at *q* = 0 or *q* = 1 (α  ≤  0.05; see Table S2 in the supplemental material). Tests for significant differences in alpha-diversity between communities were performed with *D* calculated at *q* = 1 (Table 1 and Fig. 4). Significant differences in alpha-diversity between communities from different pelagic zones were detected only in the Angola Basin (*F*_2, 8_ = 4.58; *P* = 0.047). Posthoc tests (Tukey’s honestly significant difference [HSD] test) detected significant differences in alpha-diversity between the epipelagic and bathypelagic communities in this basin (*P* = 0.039).

**TABLE 1:** *P* values for ANOVA and Tukey HSD tests comparing alpha-diversity across basins and pelagic zones^*a*^

Basin^*b*^	ANOVA *P* value^*c*^	ANOVA *F*-statistic	df	Tukey HSD comparison	Tukey HSD *P* value^*c*^
WA	0.452	0.60	1,14		
EA	0.047*	4.58	2,8	Epipelagic-bathypelagic	0.039*
				Mesopelagic-bathypelagic	0.450
				Mesopelagic-epipelagic	0.215
CM	0.414	0.74	1,8		
EM	0.052	4.39	1,16		
CS	0.231	1.55	1,17		
AB	0.131	2.29	2,17		

a Significant differences in alpha-diversity were detected only in the eastern Atlantic Ocean (α  ≤  0.05). Tukey HSD test detected a significant difference between the epipelagic (shallow-water) and bathypelagic (deep-water) communities in that basin (α  ≤  0.05).

b Abbreviations: WA, western Atlantic Ocean (Sargasso Sea); EA, eastern Atlantic Ocean (Angola); CM, central Mediterranean Sea (Libya); EM, eastern Mediterranean Sea (Egypt); CS, Caspian Sea; AB, Great Australian Bight (AB).

c Significant results are indicated by an asterisk.

10.1128/mBio.01448-20.2 FIG S2 Alpha-diversity (Hill number, *D*, *q* = 1) versus basin (group by pelagic zone). Within each basin, alpha-diversity of microbial communities was generally not significantly different by pelagic zone. Download FIG S2, DOCX file, 0.2 MB. Copyright © 2020 Miller et al. 2020 Miller et al. This content is distributed under the terms of the Creative Commons Attribution 4.0 International license.

10.1128/mBio.01448-20.3 TABLE S1 ANOVA of alpha-diversity for epipelagic and mesopelagic communities across basins. Depending on the depth to sea floor, different pelagic zones exist in a given basin. Only the epipelagic and mesopelagic zones were sampled in all basins. Therefore, only communities from those two zones were used to test for significant differences in alpha-diversity of microbial communities across basins and depth. Significant differences were detected in alpha diversity between communities across basins and pelagic zones (α ≤ 0.05). Download Table S1, DOCX file, 0.01 MB. Copyright © 2020 Miller et al. 2020 Miller et al. This content is distributed under the terms of the Creative Commons Attribution 4.0 International license.

10.1128/mBio.01448-20.4 TABLE S2 Regression of alpha-diversity (Hill number, *D*) versus sequencing depth at *q* = 0 and *q* = 1. No significant effect of sequencing depth on alpha-diversity was detected (α ≤ 0.05). Download Table S2, DOCX file, 0.01 MB. Copyright © 2020 Miller et al. 2020 Miller et al. This content is distributed under the terms of the Creative Commons Attribution 4.0 International license.

**FIG 5:**
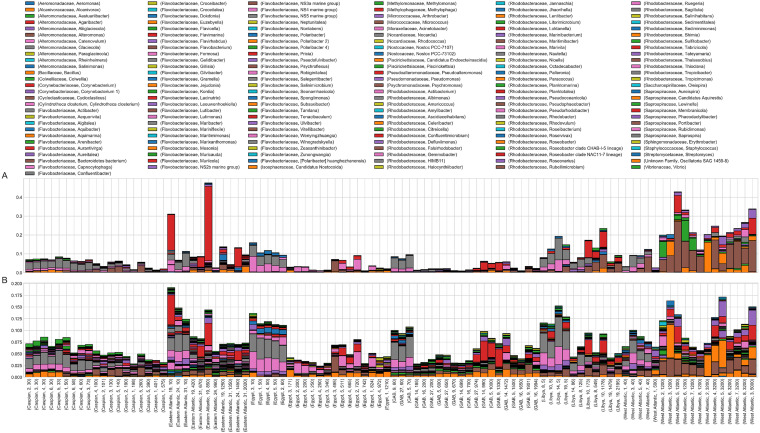
Relative abundance and diversity of hydrocarbon-degrading genera across basins. (A) Relative abundance of hydrocarbon-degrading genera across basins. The deep waters of the Sargasso Sea are enriched in potential hydrocarbon-degrading genera. Two communities from the Angola Basin were highly enriched in *Pseudoalteromonas*. (B) The number of observed OTUs for each genus is more similar across basins than the relative abundance of those genera.

Dissimilarities between microbial communities (beta-diversity) were calculated as pairwise turnover using Hill numbers *D*, *q* = [0, 1, 2]) from the rarefied OTU read counts for each community (Fig. 5). Distance-based redundancy analysis (dbRDA) was used to summarize the dissimilarity results in fewer dimensions. The dbRDA was constrained on basin and pelagic zone to capture the variance between communities based on those explanatory variables. dbRDA indicates that microbial communities from the Caspian Sea are distinct, while other microbial communities are not clearly grouped by basin of origin, which is consistent with previous reports (32). However, all microbial communities, regardless of basin, appear to be distributed along a gradient by depth. Environmental factors that were correlated with depth (i.e., increased with depth) include inorganic phosphate, nitrate, and silicate. Environmental factors that were inversely correlated with depth (i.e., decreased with depth) include nitrite, temperature, DO, and pH. These environmental factors are likely strong influences on microbial community composition.

## DISCUSSION

The primary goal of this study was to determine the relative importance of geographic location, depth, and environmental factors in shaping the taxonomic composition of these microbial communities, with particular attention to petroleum-degrading bacteria. Hydrocarbon-degrading microorganisms that might be involved in petroleum biodegradation were identified based on either association with a petroleum spill *in situ* or on experimental evidence of biodegradation of common petroleum hydrocarbons (e.g., aromatic and polycyclic aromatic hydrocarbons). Characterization of the microbial community in the Gulf of Mexico following the DWH spill provided insight about microbial taxa that are involved in petroleum biodegradation *in situ* (1). Following the well blow out, *Oceanospirillales*, putative alkane degraders, quickly dominated the community (5, 17, 25, 33). Approximately 6 weeks later, the microbial community composition had changed and was dominated by *Colwellia* and *Cycloclasticus.* When the well was closed 12 weeks later, the community composition had again shifted and was dominated by *Flavobacteria*, *Alteromonadaceae*, and *Rhodobacteraceae*, which are hypothesized to degrade high-molecular-weight organic compounds. Similar transitions have been observed in microcosm experiments, indicating that *Oceanospirillales*, *Colwellia*, and *Cycloclasticus* are enriched following amendment of seawater samples with petroleum hydrocarbons (22, 23, 34). Additionally, many microbes have been experimentally characterized for biodegradation of aromatic and/or polycyclic aromatic hydrocarbons, which are components of petroleum. For simplicity, these two groups of microbes are collectively referred to here as potential “hydrocarbon-degrading” microbial taxa, and their abundance across marine basins was investigated.

Microbes commonly such as *Oceanospirillales* that are associated with hydrocarbon biodegradation were ubiquitous across marine basins, but there was substantial variation in specific genera even within each basin. Although the precise contribution of most microbial taxa *in situ* is not experimentally verified, many microbes are commonly associated with hydrocarbon biodegradation in the literature (6). A few hydrocarbon-degrading genera (e.g., *Actinobacteria*, *Pseudomonas*, and *Rhodobacteriacea*) were common across all basins. On the other hand, a high abundance of 16S rRNA gene amplicon reads was observed for a few hydrocarbon-degrading genera in specific basins: *Pseudoalteromonas* was in highest abundance in three communities from the Angola Basin; *Alteromonas*, *Bacillus*, and *Oleibacter* were in highest abundance in the deep waters of the Sargasso Sea, and *Halomonas* was in highest abundance in the Central Mediterranean (Libya). When considered as a whole, hydrocarbon-degrading genera were in highest abundance in shallow water communities, but these may be microbes associated with metabolism of algal lipids and not petroleum hydrocarbons *per se* (Fig. 5 ). Hydrocarbon-degrading genera were present in highest abundance in the Atlantic Ocean (Sargasso Sea and Angola). The richness of hydrocarbon-degrading genera (proportional number of OTUs assigned to each genera) was more even across microbial communities. Richness of hydrocarbon degraders was highest in the Sargasso Sea, followed by the Angola Basin. However, richness of hydrocarbon degraders in the Sargasso Sea was highly variable, with no strong trend by depth.

The Oil-Spill Contingency and Response model (OSCAR) predicts the fate of crude oil in marine ecosystems (35). OSCAR calculates the first biotransformation rather than complete mineralization. In this model, hydrocarbons with similar chemical properties are grouped into categories based on boiling point differences. A fundamental assumption of the OSCAR model is that biodegradation decreases with temperature. During the DWH spill, however, microbial petroleum biodegradation exceeded OSCAR model predictions (1). It has since been shown that actual microbial biodegradation rates for some hydrocarbon groups are different than those predicted by the OSCAR model (34). It is therefore important to understand the microbial community in each marine basin in order to develop an informed response in the event of a spill.

Local environmental factors shape the community composition, and thus influence the abundance of putative and known petroleum-degrading microbes within each basin. Richness and alpha-diversity metrics attempt to quantify the overall biodiversity within a community, and higher values correspond to higher biodiversity. The Caspian Sea is the most shallow basin sampled in this study, and all of the Caspian Sea communities are distinct from communities from other basins (Fig. 5). Within the Caspian Sea, microbial communities are distributed along a gradient by depth, which is consistent with the other basins. The Sargasso Sea is the deepest basin sampled in this study, and this may explain why all of these communities clustered together apart from other basins. The remaining deep-water communities tended to cluster by basin, with communities from the epipelagic, mesopelagic, and bathypelagic zones sometimes appearing within the same cluster. Microbial communities from the Mediterranean Sea (Libya, Egypt) were similar to each other, but a few communities from the Great Australian Bight were also similar. This was surprising due to the large distance between these basins and because the Mediterranean Sea is highly trafficked and polluted, while the Great Australian Bight is relatively pristine. Overall, these results indicate that microbial communities are strongly influenced by their proximity to the surface (and perhaps the seafloor); i.e., shallow-water communities are highly similar across all basins. The deep-water communities are more strongly grouped by basin compared to the shallow-water communities. Pelagic zones are defined by discrete depths rather than environmental factors, and aside from the shallow epipelagic zone, pelagic zones may not be useful for distinguishing microbial communities.

A fundamental question for marine microbial ecology is how factors such as selection and dispersal interact to influence community composition across different biomes (31, 36, 37). The Caspian Sea is landlocked and also has the lowest salinity among the basins in this study, and communities from this basin were distinct from other basins. Unfortunately, it is not possible to distinguish how each of those factors separately contributes to the distinctiveness of the Caspian Sea communities. Dispersal may be a strong influence that acts to make communities relatively similar across basins (except the Caspian Sea), while selection induced by environmental features such as light and temperature influences communities within each basin. If dispersal of microbes across basins were not a strong force, then communities should have grouped more strongly by basin at all depth categories.

### Conclusions.

This work advances the current knowledge about the presence of microbes that are associated with hydrocarbon biodegradation in marine basins. Overall, petroleum degraders comprised a small proportion (≤20%) of the communities of the Caspian Sea, Great Australian Bight, and eastern Mediterranean, and their abundance was highly variable in the Angola Basin and central Mediterranean. On the other hand, these taxa comprised a large proportion (>20%) of the Sargasso Sea deep-water communities. The Mediterranean Sea is interesting because of the substantial variation in the abundance of petroleum-degrading genera across communities. The differences in abundance and variance between the central (Libya) and eastern (Egypt) Mediterranean Sea indicates that these are distinct habitats, despite being closely connected and showing only minor differences in environmental factors such as temperature and nutrient concentrations. These results are a snapshot of the microbial communities in these basins, and future studies should look at how microbial community composition in these basins changes over time. Models of petroleum degradation such as OSCAR that rely on environmental factors would likely predict similar outcomes for these two basins, but the differences in the microbial communities suggest that there may be important differences in petroleum biodegradation.

The abundance and diversity of petroleum-degrading genera in the Angola Basin suggests that these communities are well adapted for petroleum biodegradation. The Angola Basin also receives nutrient enrichment from the Angola-Benguela Front and Angola Dome, which will likely enhance biodegradation of petroleum hydrocarbons. The deep waters of the Angola Basin, like the Gulf of Mexico, are cold, reaching 4°C. Given the precedent for rapid biodegradation of petroleum hydrocarbons established after the DWH spill and the diversity and abundance of petroleum-degrading genera, it is likely that the Angola Basin microbial communities would rapidly degrade petroleum hydrocarbons in the event of a spill.

## MATERIALS AND METHODS

### Sample collection and environmental factors.

Water samples were collected in Niskin bottles in six marine basins. Water samples from five marine basins were collected as part of BP’s oceanographic survey in 2013 and 2014: Angola Basin, central and eastern Mediterranean Sea (Libya and Egypt, respectively), the Great Australian Bight (Australia), and the Caspian Sea (Azerbaijan). Additional water samples were collected from the Sargasso Sea in 2014. Samples from the Sargasso Sea were collected in triplicate. In total, 142 samples were obtained (98 samples after dereplication, described below). A MIDAS CTD+ profiler (Valeport Ltd., St. Peter’s Quay, UK) was attached to the sampling rosette for continuous monitoring of physical and chemical water parameters (e.g., temperature, dissolved oxygen, salinity, pH, turbidity).

*In situ* sampling of ambient seawater was conducted as follows. In the Caspian Sea and Angola Basin, ambient seawater (62 to 123 liters) was filtered at depth using a large-volume pump (McLane Research Laboratories, East Falmouth, MA). The volume of water sampled varied due to the differences in the amount of particulate matter at each sample location, which affected filtration. In Australia (GAB), Sargasso Sea, and the Mediterranean Sea, water was filtered on deck immediately following recovery. Water was filtered through a 142-mm nylon membrane with a pore size of 0.2 μm (Sterlitech, Kent, WA) and then stored at –20°C. One third of the filter was used for DNA analysis reported here.

Forty milliliters of water was fixed in 4% formaldehyde and stored at 4°C for acridine orange direct counts (AODCs). One hundred milliliters of water was frozen at –20°C for analysis of dissolved organic carbon and inorganic nutrients. Total organic carbon and total nitrogen were analyzed with a TOC-L analyzer (Shimadzu Scientific Instruments, Columbia, MD), and inorganic nutrients were analyzed with a SEAL AutoAnalyzer 3 HR (SEAL Analytical Inc., Mequon, WI). Nutrient concentrations (nitrate, nitrite, ammonia, total nitrogen, inorganic phosphate, silicate) for each sampling location were determined by the SOEST Lab at the University of Hawaii. Pairwise rank correlations between environmental features were calculated in Python using Kendall’s tau (scipy.stats.kendalltau), which accounts for tied pairs (32).

### DNA extraction and 16S rRNA gene amplicon sequencing.

Genomic DNA was extracted as described by Miller et al. (38) with modifications as described by Hazen et al. (1). DNA was cleaned using the Genomic DNA Clean & Concentrator kit (Zymo Research, Irvine, CA). Quality of extracted DNA was determined by measuring the 260/280 and 260/230 ratios on a NanoDrop spectrophotometer (Thermo Fisher Scientific, Waltham, MA). DNA concentration was determined by PicoGreen (Thermo Fisher Scientific, Waltham, MA).

The 16S rRNA gene libraries were prepared as described by Caporaso et al. (39). The V4 region of the 16S rRNA gene was amplified by PCR using Phusion DNA polymerase (Master Mix; Thermo Fisher Scientific, Waltham, MA) and universal primers 515f and barcoded 806r, which anneal to both bacterial and archaeal sequences. A 12-bp barcode index on the reverse primer enabled multiplexing samples for sequencing analysis. The 16S rRNA gene amplicons were then pooled together, and the quality and size of the amplicons were analyzed using a bioanalyzer (Agilent Technologies, Santa Clara, CA). The 16S rRNA gene libraries were sequenced using a MiSeq with a V2 kit (Illumina, San Diego, CA).

### Analysis of resulting sequence reads.

The resulting DNA sequences were analyzed using the following QIIME (v1.9) pipeline (39). The paired-end sequences were joined using fastq-join (40). The joined sequences were then demultiplexed, and sequences with a phred score below 20 were removed. Chimeric sequences were detected using UCHIME (41, 42) and removed. Sequences were clustered into operational taxonomic units (OTUs) at 97% sequence similarity using UCLUST (41) with QIIME’s “open-reference OTU picking” protocol. Taxonomy was assigned to a representative sequence from each OTU using UCLUST against the SILVA 132 QIIME compatible database (43, 44). Samples with fewer than 20,000 sequences were removed from the data set. For samples taken in triplicate, the mean read count was calculated and converted to the nearest integer; this number was used for downstream analysis. Samples were grouped by basin and pelagic zone for analysis of environmental parameters and microbial community composition.

### Alpha-diversity analysis.

Each sample was subsampled (i.e., “rarefied”) to 20,490 sequences prior to computing alpha-diversity metrics in order to control for differences in sequencing coverage across samples. Alpha-diversity was calculated in Hill numbers *D* with *q* from 0 to 3. Hill numbers are preferable to other pseudodiversity metrics (e.g., Shannon, Simpson) because they are in units of “effective” number of species, which are familiar and easy for most people to understand. As *q* increases, low-abundance OTUs are assigned less weight, and high-abundance OTUs are assigned more weight; this enables the investigation of diversity at different scales. That is, at *q* < 1, *D* favors low-abundance OTUs, while for values of *q* > 1, *D* favors dominant OTUs. At *q* = 0, *D* is simply the number of observed OTUs (richness). At *q* = 1, *D* = exp (Shannon), and at *q* = 2, *D* is equal to inverse Simpson’s index.

The sample sizes across basins and pelagic zones were inconsistent. Therefore, analysis of variance (ANOVA) was used to test for significant differences in alpha-diversity across pelagic zones only within each basin. Furthermore, any pelagic zone(s) with fewer than three samples were removed prior to statistical hypothesis testing. Results of ANOVA were considered statistically significant if α ≤ 0.05. If a significant difference was detected using ANOVA, *posthoc* tests were performed by Tukey’s honestly significant difference (HSD) test to determine which pelagic zones were significantly different, with α ≤ 0.05.

### Beta-diversity analysis.

Pairwise community dissimilarity (beta-diversity) was calculated using rarefied read counts as turnover using Hill numbers *D* with *q* from 0 to 3 (45). Turnover is the proportional difference in community composition for two communities. Varying the *q* parameter has an effect analogous to that of alpha-diversity calculated in Hill numbers. Pairwise turnover between microbial communities was calculated using the d function from the vegetarian package (v1.2). The *q* parameter for the *d* function was varied from 0 to 3 to evaluate the effect of down weighting low-abundance OTUs (as *q* increases). Hierarchical clustering and distance-based redundancy analysis (dbRDA) were performed using pairwise turnover to identify groups of communities that were similar to each other. Hierarchical clustering was performed in Python using the scipy.cluster.hierarchy.linkage method, and dbRDA was performed in R using the dbrda function from the vegan package (v2.5) (40, 46).
