# Supplementary material for: Microbial Communities across Global Marine Basins Show Important Compositional Similarities by Depth
Source: mBio. 2020 Aug 18;11(4):e01448-20. doi: 10.1128/mBio.01448-20 (PMC7439485; doi:10.1128/mBio.01448-20)
Supplement: FIG S1 [file mBio.01448-20-sf001.docx]

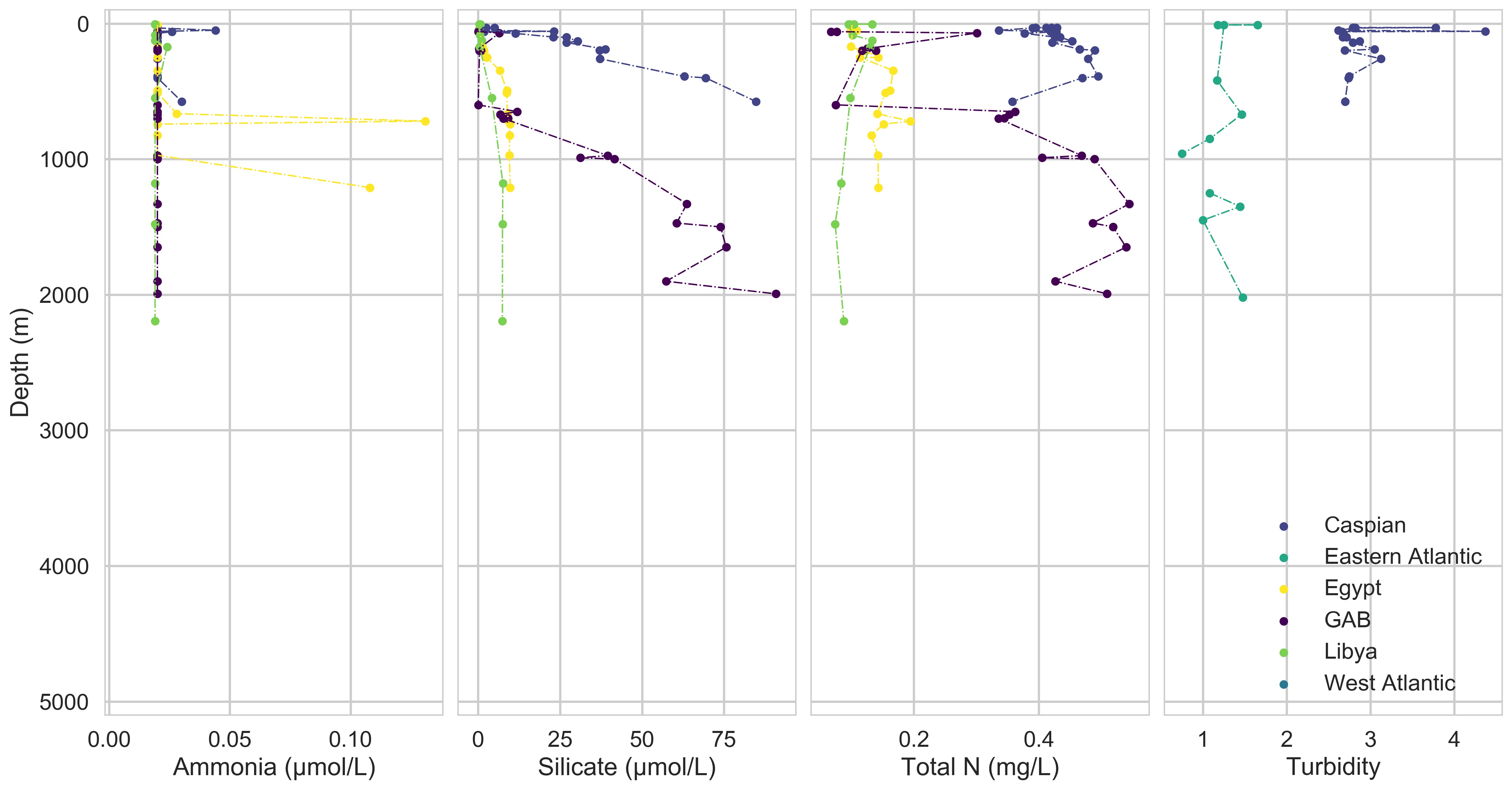


Figure S1. Supplemental depth profiles for environmental factors across basins. Data on some environmental factors was not available for all basins; no environmental data was available for the West Atlantic.
