# Supplementary material for: Microbial Communities across Global Marine Basins Show Important Compositional Similarities by Depth
Source: mBio. 2020 Aug 18;11(4):e01448-20. doi: 10.1128/mBio.01448-20 (PMC7439485; doi:10.1128/mBio.01448-20)
Supplement: FIG S2 [file mBio.01448-20-sf002.docx]

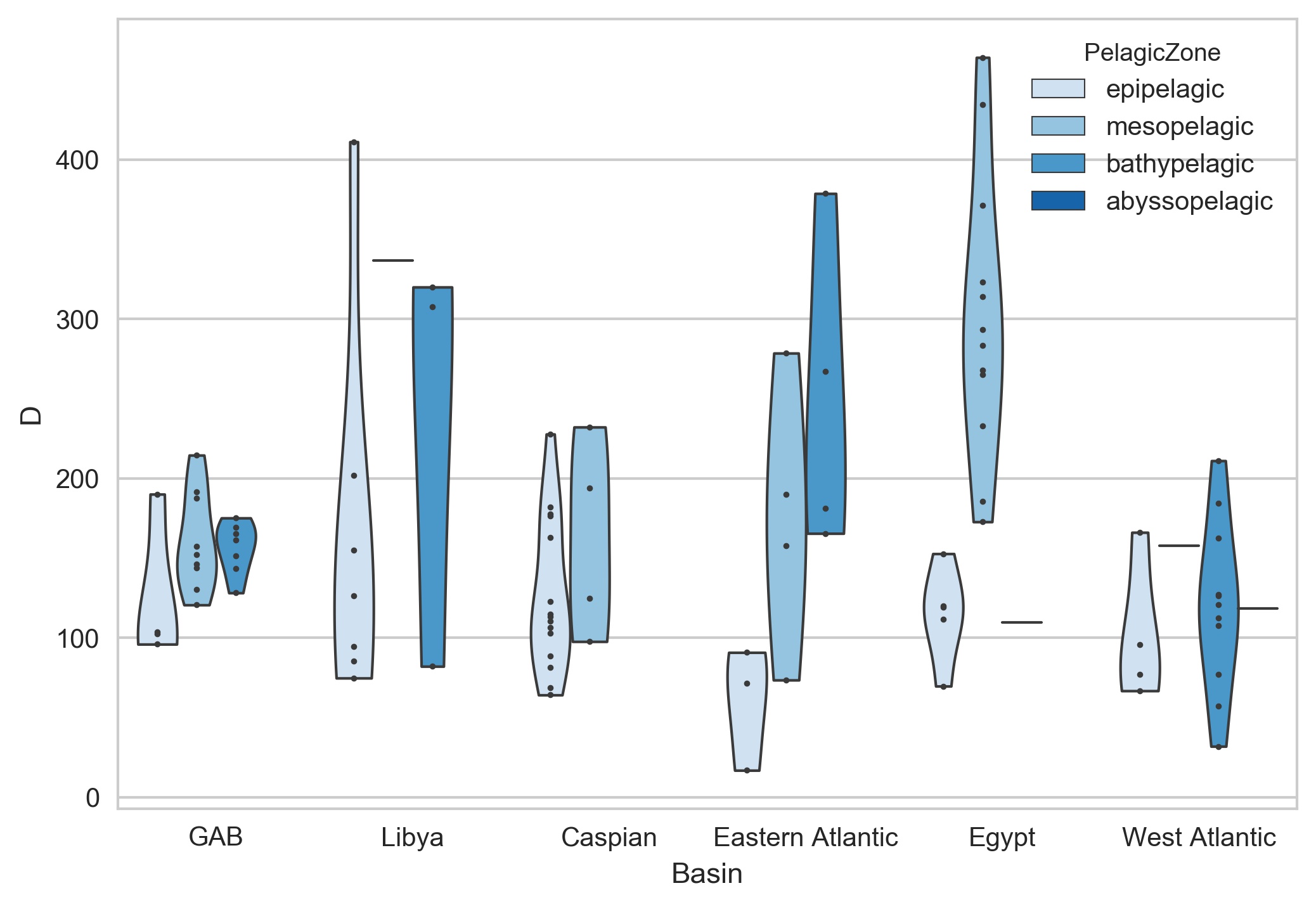


Figure S2. Alpha-diversity (Hill number, D, $q=1$) vs basin (group by pelagic zone). Within each basin, alpha-diversity of microbial communities was generally not significantly different by pelagic zone.
