# Supplementary material for: Microbial Communities across Global Marine Basins Show Important Compositional Similarities by Depth
Source: mBio. 2020 Aug 18;11(4):e01448-20. doi: 10.1128/mBio.01448-20 (PMC7439485; doi:10.1128/mBio.01448-20)
Supplement: TABLE S1 [file mBio.01448-20-st001.docx]

**Table S1.** ANOVA of alpha-diversity for epipelagic and mesopelagic communities across basins. Depending on the depth to sea floor, different pelagic zones exist in a given basin. Only the epipelagic and mesopelagic zones were sampled in all basins. Therefore, only communities from those two zones were used to test for significant differences in alpha-diversity of microbial communities across basins and depth. Significant differences were detected in alpha diversity between communities across basins and pelagic zones ($\alpha\leq0.05$).

|  | Df | Sum Sq | Mean Sq | F value | Pr(>F) |
| --- | --- | --- | --- | --- | --- |
| Basin | 5 | 375263.5 | 75052.7 | 9.42 | 8.21e-07 |
| Pelagic zone | 1 | 107847.2 | 107847.2 | 13.54 | 4.72e-04 |
| Basin:Pelagic zone | 5 | 44465.5 | 8893.1 | 1.12 | 3.60e-01 |
| Residuals | 66 | 525790.8 | 7966.5 |  |  |
