# Supplementary material for: Microbial Communities across Global Marine Basins Show Important Compositional Similarities by Depth
Source: mBio. 2020 Aug 18;11(4):e01448-20. doi: 10.1128/mBio.01448-20 (PMC7439485; doi:10.1128/mBio.01448-20)
Supplement: TABLE S2 [file mBio.01448-20-st002.docx]

**Table S2.** Regression of alpha-diversity (Hill number, $D$) vs sequencing depth at $q=0$ and $q=1$. No significant effect of sequencing depth on alpha-diversity was detected ($\alpha\leq0.05$).

| $q$ | $p$-value | $t$-statistic |
| --- | --- | --- |
| 0 | 0.21 | 1.26 |
| 1 | 0.88 | -0.15 |
